# Using EGARCH models to predict volatility in unconsolidated financial markets: the case of European carbon allowances

**DOI:** 10.1007/s13412-023-00838-5

**Published:** 2023-05-11

**Authors:** Elena Villar-Rubio, María-Dolores Huete-Morales, Federico Galán-Valdivieso

**Affiliations:** 1 Department of Applied Economics. Faculty of Economics and Business, University of Granada, Campus La Cartuja, 18071 Granada, Spain; 2 Department of Statistic and Operational Research, Faculty of Labour Sciences, University of Granada, Campus La Cartuja, 18071 Granada, Spain; 3 Department Business and Economics, University of Almeria, 04120 Almeria, Spain

**Keywords:** Volatility, Risk management, EGARCH, Carbon markets, European Union Allowances, EU ETS

## Abstract

The growing interest and direct impact of carbon trading in the economy have drawn an increasing attention to the evolution of the price of CO2 allowances (European Union Allowances, EUAs) under the European Union Emissions Trading Scheme (EU ETS). As a novel financial market, the dynamic analysis of its volatility is essential for policymakers to assess market efficiency and for investors to carry out an adequate risk management on carbon emission rights. In this research, the main autoregressive conditional heteroskedasticity (ARCH) models were applied to evaluate and analyze the volatility of daily data of the European carbon future prices, focusing on the last finished phase of market operations (phase III, 2013–2020), which is structurally and significantly different from previous phases. Some empirical findings derive from the results obtained. First, the EGARCH (1,1) model exhibits a superior ability to describe the price volatility even using fewer parameters, partly because it allows to collect the sign of the changes produced over time. In this model, the Akaike information criterion (AIC) is lower than ARCH (4) and GARCH (1,1) models, and all its coefficients are significative (*p* < 0.02). Second, a sustained increase in prices is detected at the end of phase III, which makes it possible to foresee a stabilization path with higher prices for the first years of phase IV. These changes will motivate both companies and individual energy investors to be proactive in making decisions about the risk management on carbon allowances.

## Introduction

Emissions trading is a market instrument that pursues an environmental benefit, creating an economic incentive or disincentive to reduce industrial plants emissions of polluting gases into the atmosphere, thus minimizing the associated environmental externalities. This mechanism both imposes a maximum on the levels of CO_2_ that can be emitted by industrial facilities and airlines, and it also issues allowances that concede the rights to emit a limited amount of gases, a limit which tend to be reduced over time. In addition, a market is created for the purchase and sale of those allowances, which companies must acquire according to their yearly gas emissions (otherwise, they will be punished with economic sanctions).

The European Union Emissions Trading System (EU ETS) was ambitiously designed with the aim of achieving a mature and successful system, and currently includes a total of 30 countries (27 EU countries plus Norway, Iceland and Liechtenstein^1^). The initial goal was to achieve price levels that would lead the large polluting industries, both stationary installations and the aviation sector (incorporated into the EU ETS in 2012), to consider a technological change towards a greener and more efficient economy.

**Fig. 1:**
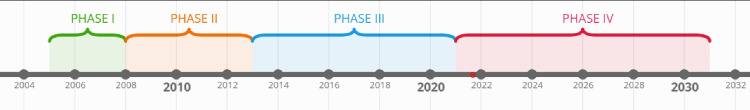
EU ETS implementation phases

Phase 3 (2013–2020), the main target of this research, began in a climate of uncertainty and pessimism, after two previous phases that were quite unstable and convulsive. The initial period or phase 1 (2005–2007) was conceived as a trial process (see timing of the phases in Fig. 1), later characterized by the existence of several errors that would lead to major improvements. First, the design of a decentralized operating scheme caused a great lack of coordination since each Member State defined its own ceilings and mode of rights allocation through the national allocation plans (NAPs). Second, the over allocation of allowances, based on estimated needs according to historical activity data, resulted in the number of allowances for the period exceeding projected emissions by some 160 million (Anderson & Di Maria 2011), which led to the price of first-period allowances falling to practically zero in 2007.

In phase 2 (2008–2012), the allocation of allowances (EUAs) continued to be decentralized but supervised by the European Commission (EC) in order to avoid over allocation. There also existed the possibility of carrying forward to phase 3 those EUAs not used in this period (known as the “banking” mechanism), aiming to reduce price volatility. Free allocation was maintained, resulting in a marginal use of allowances auctions. Although the number of allowances was reduced by 6.5%, the irruption of the economic crisis and the consequent reduction in economic and industrial activity, caused the demand for allowances to plummet, which together with the lack of unanimous agreements and delays in decision making, prolonged the negative outlook for the evolution of the EUA price, clearly resulting in a new surplus of allowances.

In phase 3 (2013–2020) some significant changes took place that led to a new twist in market operations. Among them, the following stand out: (i) instead of the previous national emission limits system, a single EU-wide emission limit applied (reduced by 1.74% per year); (ii) auctioning was the widespread method of allocating allowances (instead of free allocation), and free distribution allowances were subject to harmonized allocation rules; (iii) regulation included a higher number of sectors and gases covered; and (iv) the new entrants reserve was endowed with 300 million allowances to finance the deployment of innovative renewable energy and carbon capture and storage technologies through the NER300 program.^2^

As for the fourth phase (2021–2030), on April 8, 2018, the Directive (EU) 2018/410 (revised EU ETS Directive) entered into force, introducing the necessary changes to the ETS for compliance with the EU’s emission reduction targets under the 2030 climate and energy framework (European Commission 2020) and as part of the EU’s contribution to the implementation of the 2015 Paris Agreement (United Nations Framework Convention for Climate Change 2017). The first global stocktaking is planned for 2023. The revised Directive focus on four aspects: (i) consolidating the EU ETS as an investment driver, increasing the pace of emission allowance reductions to 2.2% per year starting in 2021; (ii) strengthening the market stability reserve mechanism, established by the EU in 2015 to reduce the surplus of allowances in the carbon market and improve the resilience of the EU ETS to future shocks; (iii) maintaining the free allocation of emission allowances as a guarantee of international competitiveness of industrial sectors exposed to a risk of carbon leakage, i.e., the risk that a company will relocate its production to countries where it is not mandatory to pay to emit greenhouse gases if it is too expensive for them to operate in Europe; and (iv) helping industrial and energy sectors to meet the innovation and investment challenges of the transition to a low-carbon economy through a range of financing mechanisms.

The recently concluded phase III (2013–2020) is the longest operational period to date, a stage in which important transformations have taken place that will provide new perspectives for the future, especially with respect to market efficiency, a necessary feature for financial markets to fulfill their primary tasks (Charles et al. 2013). Market efficiency is critical for policymakers and financial supervisors, and it is reached when information is complete, perfect, and costless, and is fully reflected in the price of the exchanged financial assets (Fama 1991). In this context, market participants are unable to consistently anticipate the movements of asset prices to obtain returns above average, and thus, market prices reflect the real behavior and interaction of the forces of supply and demand (preventing price manipulations that benefit some investors and harm the rest). In financial markets, lower levels of volatility imply higher levels of market efficiency. In fact, volatility is a fundamental and intrinsic characteristic of financial series and describes price fluctuations based on their degree of variation over time. Volatility, which is frequently measured by the variance or the standard deviation of returns, is also relevant for investors, so as they can estimate the risk-reward profile of their investments, adjust their portfolios, and even for the valuation of financial options through the Black–Scholes formula.

Volatility implies variability and instability in the trajectory of the financial series and is usually not constant. For this reason, classical models with homoscedastic variance are not appropriate, and it is necessary to apply autoregressive conditional heteroskedasticity econometric models, that is, ARCH-type models. The ARCH model has the advantage of considering conditional volatility, but the disadvantage that the number of parameters to be estimated is usually very high (and therefore, worse fits are usually obtained) in this type of financial series. The GARCH model has an advantage over the ARCH model, since the conditional variance not only depends on the squares of the observations, but also depends on the conditional variances of previous periods. However, it does not include the sign (positive or negative) of these variations. This inconvenience is solved by the EGARCH model, by allowing to identify the positivity or negativity in the price fluctuations.

Given the scarcity of research about risk in carbon markets (Zhu et al. 2019), our main aim is to explore and analyze the market volatility of the European CO2 allowances future market by applying these three ARCH-type models (ARCH, GARCH, and EGARCH).

After the introduction, the rest of the article is divided into four additional sections. Section 2 details some previous contributions regarding the application of GARCH and EGARCH models in markets with high price volatility. Section 3 includes the data sample and a brief description of the EGARCH methodology. Section 4 shows the main results derived from the empirical analysis, and finally, Sect. 5 exhibits the main conclusions, limitations, and future lines of research.

## Background

The European carbon market has attracted academic attention since its inception, given its novelty (acting as a testing laboratory for all the countries and economic regions aiming to create a similar mechanism, such as China) and its worldwide implications for a greener and sustainable economy. Risk management is a major issue for academics, practitioners, and policymakers in the carbon market. Researchers have tackled this question measuring risk using different approaches, particularly volatility and the value at risk (VaR) method. This method only focuses on the probability of a maximum loss in a time interval, given a certain degree of confidence (Sheng et al. 2021), and has been applied to the study of carbon markets (Zhu et al. 2019). About the former, researchers have put a special emphasis in volatility spillovers between carbon markets and other related such as oil (Do et al. 2020; Oikonomikou 2018; Y. J. Zhang and Sun 2016), the presence of persistent volatility asymmetry (Bentes 2018; Olbrys & Majewska 2017), and volatility clustering, that is (and as any other tradeable asset), the price of carbon allowances is exposed to periods with high volatility and low volatility (Aliyev et al. 2020).

In this regard, the family of autoregressive conditional heteroskedasticity models (ARCH) (Bollerslev 1986; Engle 1982) has proven to be the best methods to measure volatility. Among the wide variety of extensions, the generalized ARCH (or GARCH) subtype stands out in recent literature on financial markets dynamics for developed regions such as the major European stock markets of the UK, France, and Germany (Olbrys & Majewska 2017); South Europe and Ireland (Bentes 2018); the USA (Aliyev et al. 2020); and China (Do et al. 2020), but also for less developed stock markets such as Pakistan (Mohsin et al. 2020) or Ghana (Omari-Sasu et al. 2015). GARCH models have also been applied to the analysis of exchange rate markets volatility (Hung 2021).

Volatility on energy markets has been modelized using GARCH-type models in the Portuguese and Spanish electricity markets (Macedo et al. 2020; J. Zhang and Tan 2013); US crude oil markets (Lin et al. 2020); crude oil, natural gas, and electricity in the USA (Efimova and Serletis 2014); crude oil and new energy markets in China (Chen et al. 2020); or European crude oil prices (Maraqa and Bein 2020). Within the scope of CO_2_ prices in the European market, various studies have been carried out on European carbon prices and coal, natural gas, and Brent oil prices (Y. J. Zhang and Sun 2016; Zhu et al. 2019), CO_2_ emission allowances in EU-ETS phase II and phase III (Galán-Valdivieso et al. 2018), the interactions between EU Allowances and Certified Emission Reductions (CERs) in the EU-ETS (Koop and Tole 2013), or the relevance of traders’ behavior (Wang et al. 2019).

## Data and methods

### Data sample and description

**Fig. 2:**
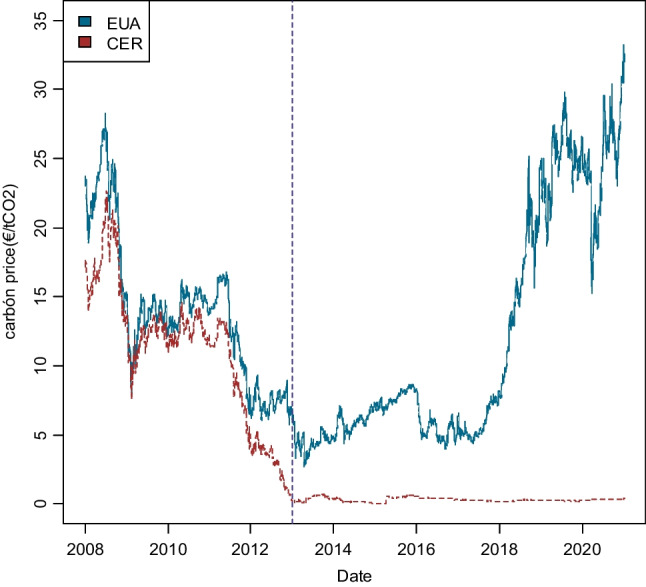
CO_2_ emission allowances (EUA) and certified emission reduction (CER) prices. From 2 January 2018 to 31 December 2020 (phase II and III)

Data used for the analysis were retrieved from the Thomson Reuters Eikon (Refinitiv) database and correspond to daily quotations of EUAs future contracts on the main organized market, the European Climate Exchange (ECX), based in London and integrated into the ICE (Intercontinental Exchange), which channels 95.2% of global EUA and CER transactions. Figure 2 shows the evolution of the daily prices of both EUAs and CERs in the two main phases of operation of the EU ETS (phase II: 2008–2012 and phase III: 2013–2020).

As can be noted, the evolution of CERs (CO_2_ credits from investments in clean mechanisms in developing countries) is similar to the EUAs’ prices until the end of 2011, but from 2012 onwards, there is a clear break in the trend of both series, where the price of CERs falls from approximately 5 euros per ton to values below 1 euro in 1 year, and close to zero from that year on. The collapse in the price of carbon credits is due to the low level of operations with these assets since the EC imposed ceilings on their use, in accordance with the principle of supplementarity, according to which the use of carbon credits from reduction projects must in any case be supplementary to the measures applied internally by the Member States. This situation generated a clear imbalance between supply and demand, with an oversupply of credits that would exceed 5000 million tons. This, together with the lack of commitment of the signatories of the Kyoto Protocol with this instrument, has led to great uncertainty about its future, as it is unable to fulfill the functions for which it was designed.

As regards the behavior of the EUA price, it was consistent with the imbalance between supply and demand in phase 2, as detailed above. After reaching a peak of 30 €/t in 2008, the price fell to 12–14€/t, remaining in this range until mid-2011, when it began to decline again to end phase 2 below €7/t. The economic recession generated an oversupply of allowances, which, together with the use of international credits (clean development mechanisms and joint implementation), generated an accumulation of unconsumed allowances of close to 2 billion at the end of 2012.

Thus, phase III was born with the problem of excess EUAs left over from phase 2, which led the price of EUA to free fall, with consecutive reductions in its price in the first weeks of up to 18%, dropping below 4 euros per ton of CO_2_. Two main reasons caused this situation: a large supply and the inexistence of counterpart demand from the European industry (with no need at that time to resort to auctions, still affected by the economic crisis) and the growing penetration of renewable energies in the electricity sector and the increase in investment in energy efficiency. Considering that the oversupply of rights is structural, and foreseeably lasting in time, as an emergency measure, the European Parliament and the Council approved in December 2013 different measures: postponing the auctioning of 900 million allowances to 2019–2020, and the creation of a market stability reserve to neutralize the negative impacts of the existing allowance surplus and improve the system’s resilience to future shocks.

In January 2016, the EU ETS suffered another major shock marked by the announcement of the UK referendum, which caused the price of CO_2_ to plummet to a 3-year low of below €5 per ton. While other markets, such as the financial market, were recovering, the carbon market extended its losses even further. The year 2017 was marked by successive rebounds that ended with an average price of €5.8 per EUA. It is worth noting the significant increase in the price of emission allowances that occurred throughout 2018, rising more than threefold from €7 to €25 per ton at the end of the year, justified by the speculative motive that led agents to buy a large quantity of allowances in the hope of selling them more expensively in the future in the expectation that Europe would raise the minimum sale price. The year 2019, despite the successive price rises and falls, was characterized by higher prices, staying on average at around €25 per ton. The lower relative price of coal, compared to natural gas, led to major purchases of EUA in advance of a foreseeably price increase of electricity.

There is a clear turning point in 2020, marked by a significant drop in mid-March as the market’s response to the health crisis triggered by COVID-19. The price of CO_2_ allowances dropped from €24.5 per ton of CO_2_ at the beginning of the year to reach its lowest peak on March 18, 2020 (days after the state of alarm was decreed in many EU countries), at a price of €15.23 per ton, placing the EUAs quotation at the lowest price since 2018. This negative response from the energy and financial markets was the reflection of anticipated forecasts of the loss of wealth and employment resulting from the containment measures necessary to control the pandemic, with the consequent paralysis of an important part of the industrial sector. For instance, electricity demand in Spain decreased by 5.6% in 2020 as a result of the pandemic, leading to a 27.8% reduction in CO_2_ equivalent emissions associated with electricity production.

During the second and third quarters of 2020, the price of EUAs rebounded according to the different worldwide surges caused by the impact of COVID-19, closing the year with a climate of optimism due to the imminent start of vaccination to stop the pandemic, which was reflected in a rise in prices, reaching 33.2 euros per ton on December 28, 2020, the highest since the EU ETS began to operate.

As described, both external and internal issues have shaped the trend of EUAs prices, resulting in large fluctuations hard to anticipate for investors (Alberola et al. 2008; Y. J. Zhang and Sun 2016). As a “young” market, the European carbon market must face internal issues that are being solved by the European authorities, in search for a stable and efficient market. This is the reason behind exploring its volatility behavior: determining if, despite the external shocks, this market is close to maturity or new measures are to be taken.

### Methods

Autoregressive conditional heteroskedastic (ARCH) model proposed by Engle (1982, 1983) has been widely used to analyze and forecast economic or financial time series characterized by periods of high or low volatility and significant kurtosis. Bollerslev (1986) proposed the generalized autoregressive conditional heteroskedastic (GARCH) model, which recognized the difference between the unconditional and the conditional variance, allowing the latter to change over time as a function of past errors. This generalized model provides a longer memory and a more flexible lag structure (Brockwell and Davis 1996). However, the GARCH model has the disadvantage that conditional variance depends on magnitude of delayed innovations, but not of their sign. This problem was solved with the exponential generalized autoregressive conditional heteroskedastic (EGARCH) model (Nelson 1991) by introducing a measure of the sign of innovations. While the starting point of GARCH models is that positive and negative error terms have a symmetric effect on the volatility, previous research has shown that this effect is in fact asymmetric in financial time series, due to market imperfections such as transaction costs (Aliyev et al. 2020) and the different response of investors to good and bad news (Barberis et al. 1998; Bentes 2018). Thus, EGARCH models are probably more suitable to analyze financial time series.

Let the series ${y}_{t}$, in the ARCH (q) model, be ${y}_{t}={\varepsilon }_{t }\cdot {\sigma }_{t}$, where ${\sigma }_{t}$ is the volatility and ${\varepsilon }_{t }$ i.i.d. white noise with zero mean and finite variance. The conditional variance of the series at each time $V\left({y}_{t}|{y}_{t-1}\right)={\sigma }_{t}^{2}$ is characterized by the following autoregressive process:

$${\sigma }_{t}^{2}={\alpha }_{0}+\sum_{i=1}^{q}{\alpha }_{i}\cdot {y}_{t-i}^{2}$$

${{\alpha }_{i}\ge 0 }_{i=1\cdots q} , {\alpha }_{0}>0$ where ${\alpha }_{0}$ is the minimal value observed of conditional variance (Bera & Higgins 1993). This ARCH model is the starting point for the temporal analysis of financial series in which the variability is not constant. The behavior of this type of series is characterized by exhibiting periods of sudden changes (higher volatility) and others full of stability, with hardly any changes (lower volatility). In general, we assume that it depends on *q* lags in the observed values, which is usually very large in this type of series and therefore requires estimating a large number of parameters. For all these reasons, generalizations such as the GARCH model arise, in which conditional variability also depends on its past values (in general, on *p* past values).

The GARCH (*p*,*q*) model adds a term that allows us to assume that the conditional variance also depends on its past observations:

$${\sigma }_{t}^{2}={\alpha }_{0}+\sum_{i=1}^{q}{\alpha }_{i}\cdot {y}_{t-i}^{2}+\sum_{i=1}^{p}{\beta }_{i}\cdot {\sigma }_{t-i}^{2}$$

where

$${{\alpha }_{i}\ge 0 }_{i=1\dots q} , {{\beta }_{i}\ge 0 }_{i=1\dots p} , {\alpha }_{0}>0, p\ge 0, q>0, \sum_{i=1}^{q}{\alpha }_{i}+\sum_{i=1}^{p}{\beta }_{i}<1$$

to ensure stationarity and a conditional variance that is strictly positive.

In both models, the conditional variance is linear in lagged and only collect the magnitude of the time changes but not the positivity or negativity. The EGARCH model solves this inconvenience. In EGARCH (*p*,*q*) model, the specification for the conditional variance is as follows:

$${ln \sigma }_{t}^{2}={\alpha }_{0}+\sum_{i=1}^{q}\left({\alpha }_{i}\cdot {y}_{t-i}+{\gamma }_{j}\left(\left|{y}_{t-i}\right|-E\left|{y}_{t-i}\right|\right)\right)+\sum_{i=1}^{p}{\beta }_{i}\cdot {ln \sigma }_{t-i}^{2}$$

where the coefficient ${\alpha }_{i}$ captures the sign effect and ${\gamma }_{j}$ the size effect. The exponential operator to get conditional volatility guarantees its positivity, so no restrictions are required, for conditions’ volatility is positive (McAleer and Hafner 2014).

The time series analysis and estimated models by maximum likelihood estimation were performed with R software (R Development Core Team 2020), specifically *forecast* (Hyndman et al. 2020), *fGarch* (Wuertz et al. 2020) and *rugarch* (Ghalanos 2020) packages.

## Results

### Descriptive analysis of CO_2_ emission allowances

Data is transformed, as usual in finance, using the natural logarithm of the daily returns, defined as:

$${y}_{t}=\mathrm{Ln }\left(\frac{{P}_{t+1}}{{P}_{t}}\right)$$

**Fig. 3:**
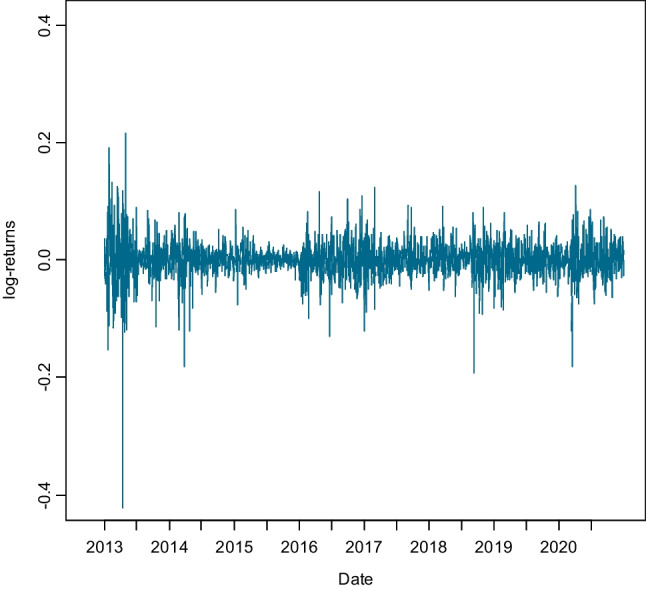
Log-returns CO_2_ emission allowances EUAs from 2 January 2013 to 31 December 2020 (phase III)

Figure 3 shows the log-returns of carbon EUAs’ future contracts. The second half of April 2013, in the midst of the economic crisis, began with a sharp fall, the most significant in all the years the market has been operating, with the price of EUA falling by 35%, close to €3/ton, reflecting a marked volatility. The large drop in the price undoubtedly had a negative impact on investment and the reactivation of the economy. The process of determining the price of the EUA is governed, like any other good or service in the economy, by the law of supply and demand, which means that an increase in the demand for EUA (ceteris paribus) will lead to an upward trend in prices, and vice versa. These changes in demand can be determined by various factors (Erias-Rey and Dopico-Castro 2011) such as variations in the prices of other energy markets, such as oil or renewable energies, the cheaper the latter are, the lower the use of EUA will fall and thus its price; phases of economic crisis or growth, which will lead to variations in the levels of industrial production and thus changes in the demand for EUA; the levels of penalties for non-compliance, which will make the purchase of rights more or less profitable; the possibility of new agents participating in the market; and exogenous factors such as the weather, which has a direct influence on the possibility of using renewable energies (solar, wind, etc.), which will undoubtedly affect the demand for rights.

The returns appear to fluctuate around a constant level, but exhibit volatility clustering. The original series is not stationary, but the series of log-returns shows low structure in the mean. Log-returns show great variability with some peaks indicating the presence of heteroskedasticity, both positive and negative. Taking this feature into account, ARCH-type models are advisable, which assume that the conditional variance depends on the past with autoregressive structure (Bollerslev et al. 1992), its generalization in the GARCH model or its exponential variant EGARCH.

**Fig. 4:**
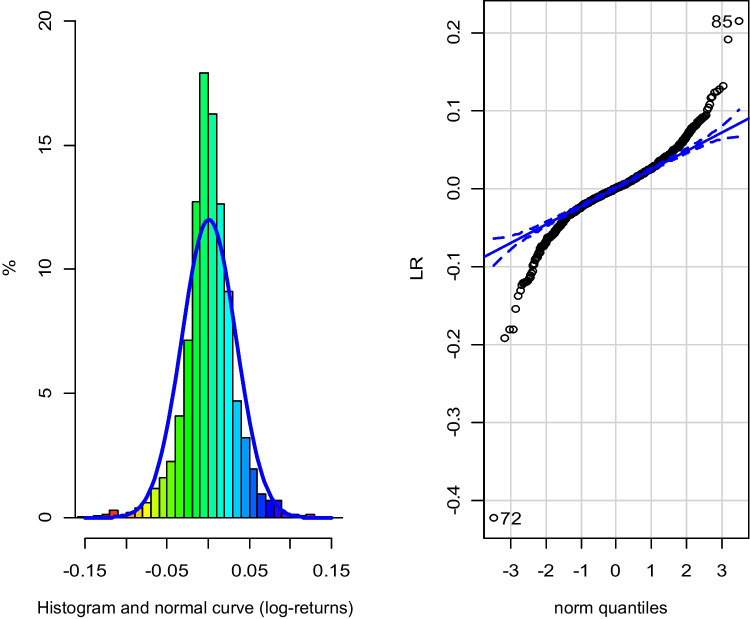
Histogram and Q-Q plot of log-returns

The great advantage of the EGARCH model is that it allows to reflect how positive and negative changes in the series affect volatility, which is not the case in the GARCH model. The conditional variance of the GARCH model is a function of the square of the past innovations, so it does not collect positive or negative changes. Regarding the normality of the log-returns (Fig. 4), the skewness coefficient is − 1.1566, which is not excessively high, but the kurtosis is equal to 16.6511, which is quite far from normal behavior. The Kolmogorov–Smirnov test indicates that log-returns cannot be considered normal (*p* < 0.0001).

We used Lagrange multiplier ARCH test (Engle 1982) to test the null hypothesis of adequately fitted ARCH type process (Fisher and Gallagher 2012), asymptotically distributed as χ^2^ under the null hypothesis of no autocorrelation in the squared residuals. This test indicates that the use of ARCH-type models is appropriate for various lags (*p* < 0.0001). The partial autocorrelation function also shows significant correlations in lags two and four.

**Fig. 5:**
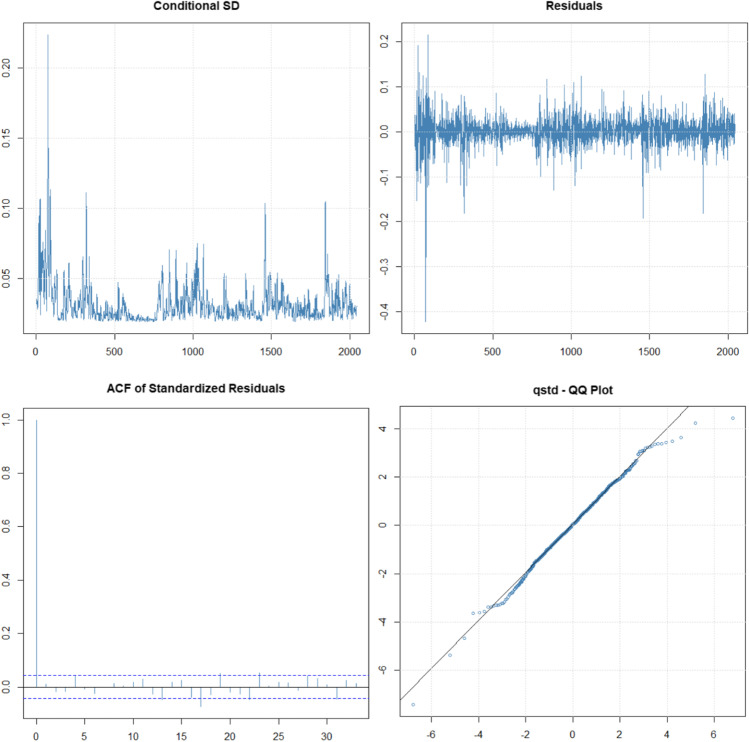
Volatility, residuals, and autocorrelation function of standardized residuals and QQ plot of standardized residuals. ARCH (4) model

The mean has not been included in the models, since it is not significant and close to zero, and the conditional distribution that bests fits is the *t*-Student. The results of the ARCH (4) model graphically indicate a good fit in this model (Fig. 5 and Table 1). There are no strange behaviors of the residuals, the ACF is significant for lag = 0, and the QQ plot indicates normality of the residuals. However, in ARCH model, often we must consider many parameters or lags to explain the volatility. In our analysis, the LM-ARCH test indicates that we need four lags in ARCH model. This problem can be solved considering the GARCH model (Zivot 2009).

**Table 1:** Estimates parameters of models and goodness of fit

*Model*	${\alpha }_{0}$	${\alpha }_{i}$	${\beta }_{i}$	${\gamma }_{i}$	AIC	Log-lik	LM arch test
*ARCH (4)*	0.0003^***^ *(2·10*^*−16*^*)*	0.1806^***^ *(2·10*^*−5*^*)* 0.1986^***^ *(2·10*^*−5*^*)* 0.2438^***^ *(2·10*^*−6*^*)* 0.1623^***^ *(9·10*^*−5*^*)*			− 4.3371	4438.57	0.0665
*GARCH (1,1)*	0.00001^*^ *(0.0137)*	0.0981^***^ *(2·10*^*−9*^*)*	0.8991^***^ *(2·10*^*−16*^*)*		− 4.3797	4480.09	0.8474
*EGARCH (1,1)*	− 0.1550^***^ *(2·10*^*−16*^*)*	− 0.0372^*^ *(0.0179)*	0.9782^***^ *(2·10*^*−16*^*)*	0.2133^***^ *(2·10*^*−16*^*)*	− 4.3842	4485.66	0.8065

The table shows the estimated value for each parameter and below, in italics, the *p*-value of the significance test

**Fig. 6:**
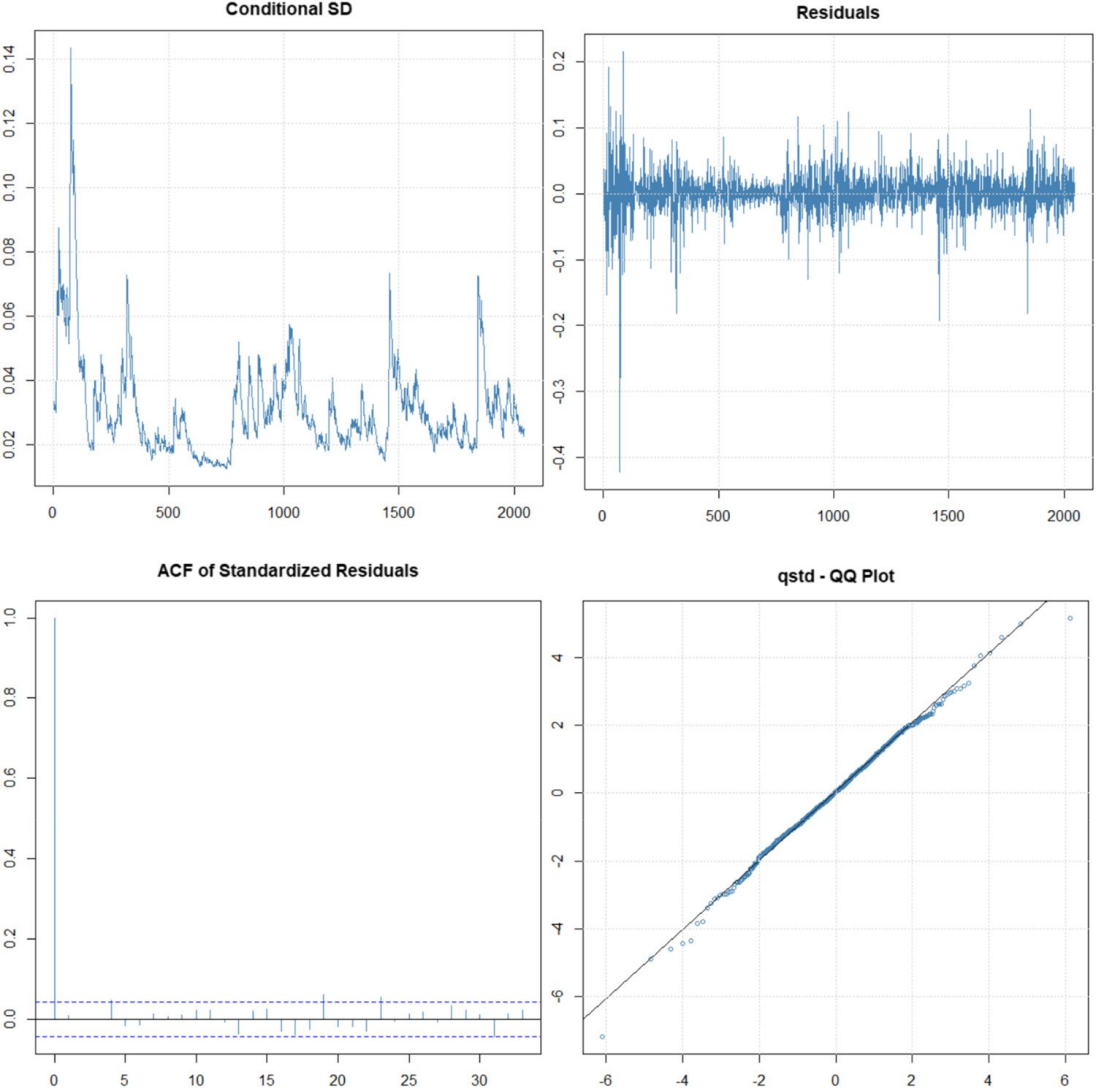
Volatility, residuals, and autocorrelation function of standardized residuals and QQ plot of standardized residuals. GARCH (1,1) model

Considering the GARCH model, the best fit is GARCH (1,1). We reduced the number of parameters with respect to ARCH model, improving the AIC/BIC test (Table 1). As previously mentioned, this is a great advantage of the GARCH model: the reduction of the parameters. Goodness of fit of this model can also be seen graphically (Fig. 6). Weighted Ljung-Box test on standardized squared residuals confirms its randomness.

**Fig. 7:**
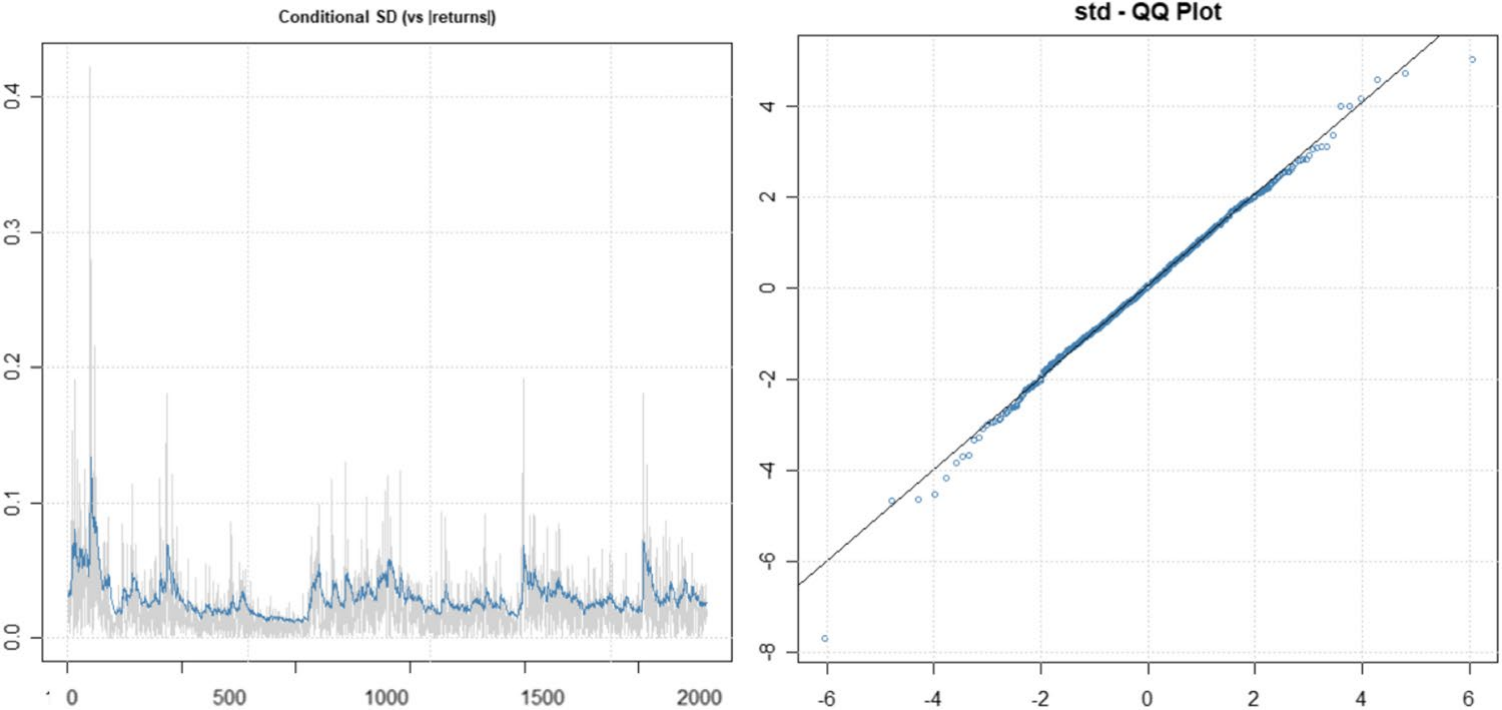
Volatility/absolute values of log-returns and QQ plot of residuals. EGARCH (1,1) model

The best EGARCH model is EGARCH (1,1) whose estimated parameters are significant and improves the log-likelihood (Table 1), confirming results from previous literature (Mohsin et al. 2020). We can also graphically observe the correct behavior of the residuals (Fig. 7).

In short, EGARCH is the best model to fit the log-returns of CO_2_ emission allowances: reduces the number of parameters compared to other models, improves the model log-likelihood, and ensures the good behavior of the residuals. These results are in line with previous research, confirming that EGARCH estimations outperform other models (Hung 2021).

## Conclusions

The recent experience of operating the EU ETS has shown that one of the greatest difficulties faced in the carbon markets is the adjustment of the optimal supply of allowances so as to ensure that the market efficiently prices the carbon emission rights. The evolution of the price of carbon allowances show that the market is already responding to the reforms introduced to strengthen the price signal, which is reflected in the consolidation and stability of the market at the end of phase III. The expected rise in the price of CO_2_ rights will undoubtedly keep affecting the price of electricity on the wholesale market, with the consequent microeconomic impact on inflation and household spending.

In this research, the modelization of volatility using EGARCH (1,1) models exhibits a greater ability to describe market behavior. These results can help policymakers to analyze the effectiveness of their legislations towards a more efficient carbon market, but also are useful results for investors and firms who must manage their risk regarding the purchase or sale of carbon emission rights. Future lines of research could focus on the aviation industry (because of its particular characteristics) or applying additional methodologies, such as the value at risk (VaR) to enlarge the applicability of the EGARCH models.

## Footnotes

1 As of January 1, 2021, and as a consequence of Brexit, installations located in the United Kingdom (UK) ceased to be part of the EU ETS, although the compliance cycle obligations corresponding to 2020 were to be fulfilled within the first months of 2021.

2 The NER 300 program took its name from the sale of 300 million emission allowances from the new entrants’ reserve (NER). Funds received from the sales (about 2 billion euros) were distributed to different selected projects.

## References

[CR1] Alberola E, Chevallier J, Chèze B (2008). Price drivers and structural breaks in European carbon prices 2005–2007. Energy Policy.

[CR2] Aliyev F, Ajayi R, Gasim N (2020). Modelling asymmetric market volatility with univariate GARCH models: evidence from Nasdaq-100. J Econ Asymmetries.

[CR3] Anderson B, Di Maria C (2011). Abatement and allocation in the pilot phase of the EU ETS. Environ Resour Econ.

[CR4] Barberis N, Shleifer A, Vishny R (1998). A model of investor sentiment. J Financ Econ.

[CR5] Bentes SR (2018). Is stock market volatility asymmetric? A multi-period analysis for five countries. Physica A.

[CR6] Bera AK, Higgins ML (1993). Arch models: properties, estimation and testing. J Econ Surv.

[CR7] Bollerslev T (1986). Generalized autoregressive conditional heteroskedasticity. J Econ.

[CR8] Bollerslev T, Chou RY, Kroner KF (1992). ARCH modelling in finance: a review of the theory and empirical evidence. J Econ.

[CR9] Brockwell PJ, Davis RA (1996). Introduction to time series and forecasting.

[CR10] Charles A, Darné O, Fouilloux J (2013). Market efficiency in the European carbon markets. Energy Policy.

[CR11] Chen Y, Zheng B, Qu F (2020). Modeling the nexus of crude oil, new energy and rare earth in China: an asymmetric VAR-BEKK (DCC)-GARCH approach. Resour Policy.

[CR12] Do A, Powell R, Yong J, Singh A (2020). Time-varying asymmetric volatility spillover between global markets and China’s A, B and H-shares using EGARCH and DCC-EGARCH models. N Am J Econ Financ.

[CR13] European Commission (2020) 2030 climate & energy framework. https://ec.europa.eu/clima/policies/strategies/2030_en. Accessed 31 Jul 2021

[CR14] Efimova O, Serletis A (2014). Energy markets volatility modelling using GARCH. Energy Econ.

[CR15] Engle RF (1982). Autoregressive conditional heteroscedasticity with estimates of the variance of United Kingdom inflation. Econometrica.

[CR16] Engle RF (1983). Estimates of the variance of U.S. inflation based upon the ARCH model. J Money Credit Bank.

[CR17] Erias-Rey A, Dopico-Castro JÁ (2011). Los mercados de carbono en la Unión Europea: Fundamentos y proceso de formación de precios. Revista Galega De Economía.

[CR18] Fama EF (1991). Efficient capital markets: II. J Financ.

[CR19] Fisher TJ, Gallagher CM (2012). New weighted portmanteau statistics for time series goodness of fit testing. J Am Stat Assoc.

[CR20] Galán-Valdivieso F, Villar-Rubio E, Huete-Morales MD (2018). The erratic behaviour of the EU ETS on the path towards consolidation and price stability. Int Environ Agreements: Polit Law Econ.

[CR21] Ghalanos A (2020) Rugarch: univariate GARCH models. (R package version 1.4-9.)

[CR22] Hung NT (2021). Volatility behaviour of the foreign exchange rate and transmission among central and eastern European countries: evidence from the EGARCH model. Glob Bus Rev.

[CR23] Hyndman R, Athanasopoulos G, Bergmeir C, Caceres G, Chhay L, O'Hara-Wild M, Petropoulos F, Razbash S, Wang E, Yasmeen F (2020) forecast: forecasting functions for time series and linear models. (R package version 8.21.). Retrieved from https://pkg.robjhyndman.com/forecast/

[CR24] Koop G, Tole L (2013). Modeling the relationship between European carbon permits and certified emission reductions. J Empir Financ.

[CR25] Lin Y, Xiao Y, Li F (2020). Forecasting crude oil price volatility via a HM-EGARCH model. Energy Econ.

[CR26] Macedo DP, Marques AC, Damette O (2020). The impact of the integration of renewable energy sources in the electricity price formation: is the merit-order effect occurring in Portugal?. Util Policy.

[CR27] Maraqa B, Bein M (2020) Dynamic interrelationship and volatility spillover among sustainability stock markets, major European conventional indices, and international crude oil. Sustainability 12(9):3908. 10.3390/su12093908

[CR28] McAleer M, Hafner CM (2014). A one line derivation of EGARCH. Econometrics.

[CR29] Mohsin M, Naiwen L, Zia-UR-Rehman M, Naseem S, Baig SA (2020). The volatility of bank stock prices and macroeconomic fundamentals in the Pakistani context: an application of GARCH and EGARCH models. Oeconomia Copernicana.

[CR30] Nelson DB (1991). Conditional heteroskedasticity in asset returns: a new approach. Econometrica.

[CR31] Oikonomikou LE (2018). Modeling financial market volatility in transition markets: a multivariate case. Res Int Bus Finan.

[CR32] Olbrys J, Majewska E (2017). Asymmetry effects in volatility on the major European stock markets: the EGARCH based approach. Quant Finan Econ.

[CR33] Omari-Sasu AY, Frempong NK, Boateng MA, Boadi RK (2015). Modeling stock market volatility using GARCH approach on the Ghana stock exchange. Int J Bus Manag.

[CR34] R Core Team (2020) R: a language and environment for statistical computing. R Foundation for Statistical Computing (version 3.6.3.). Vienna, Austria. Retrieved from https://www.R-project.org/

[CR35] Sheng C, Zhang D, Wang G, Huang Y (2021). Research on risk mechanism of China’s carbon financial market development from the perspective of ecological civilization. J Comput Appl Math.

[CR36] United Nations Framework Convention for Climate Change (2017) Paris agreement - status of ratification. http://unfccc.int/paris_agreement/items/9444.php. Accessed 25 Jun 2021

[CR37] Wang J, Gu F, Liu Y, Fan Y, Guo J (2019). Bidirectional interactions between trading behaviors and carbon prices in European Union emission trading scheme. J Clean Prod.

[CR38] Wuertz D, Setz T, Chalabi Y, Boudt C, Chausse P, Miklovac M (2020) fGarch: rmetrics-autoregressive conditional heteroskedastic modelling. (R package version 3042.83.2.). Retrieved from https://cran.r-project.org/package=fGarch

[CR39] Zhang YJ, Sun YF (2016). The dynamic volatility spillover between European carbon trading market and fossil energy market. J Clean Prod.

[CR40] Zhang J, Tan Z (2013). Day-ahead electricity price forecasting using WT, CLSSVM and EGARCH model. Int J Electr Power Energy Syst.

[CR41] Zhu B, Ye S, He K, Chevallier J, Xie R (2019). Measuring the risk of European carbon market: an empirical mode decomposition-based value at risk approach. Ann Oper Res.

[CR42] Zivot E (2009). Practical issues in the analysis of univariate GARCH models. Handbook of Financial Time Series.

